# Protocols for Endovascular Stroke Treatment Diminish the Weekend Effect Through Improvements in Off-Hours Care

**DOI:** 10.3389/fneur.2018.01106

**Published:** 2018-12-18

**Authors:** Scott B. Raymond, Feras Akbik, Christopher J. Stapleton, Brijesh P. Mehta, Ronil V. Chandra, Roberto G. Gonzalez, James D. Rabinov, Lee H. Schwamm, Aman B. Patel, Joshua A. Hirsch, Thabele M. Leslie-Mazwi

**Affiliations:** ^1^Department of Neurosurgery, Massachusetts General Hospital, Boston, MA, United States; ^2^Department of Radiology, Massachusetts General Hospital, Boston, MA, United States; ^3^Department of Neurology, Massachusetts General Hospital, Boston, MA, United States; ^4^Department of Neuroendovascular Surgery, Memorial Healthcare System, Hollywood, FL, United States; ^5^Interventional Neuroradiology, Monash Health, Melbourne, VIC, Australia

**Keywords:** ischemic stroke, weekend effect, protocol, thrombectomy, neuroendovascular

## Abstract

**Introduction:** The weekend effect is a well-recognized phenomenon in which patient outcomes worsen for acute strokes presenting outside routine business hours. This is attributed to non-uniform availability of services throughout the week and evenings and, though described for intravenous thrombolysis candidates, is poorly understood for endovascular stroke care. We evaluated the impact of institutional protocols on the weekend effect, and the speed and outcome of endovascular therapy as a function of time of presentation.

**Method:** This study assesses a prospective observational cohort of 129 consecutive patients. Patients were grouped based on the time of presentation during regular work hours (Monday through Friday, 07:00–19:00 h) vs. off-hours (overnight 19:00–07:00 h and weekends) and assessed for treatment latency and outcome.

**Results:** Treatment latencies did not depend on the time of presentation. The door to imaging interval was comparable during regular and off-hours (median time 21 vs. 19 min, respectively, *p* < 0.50). Imaging to groin puncture was comparable (71 vs. 71 min, *p* < 1.0), as were angiographic and functional outcomes. Additionally, treatment intervals decreased with increased protocol experience; door-to-puncture interval significantly decreased from the first to the fourth quarters of the study period (115 vs. 94 min, respectively, *p* < 0.006), with the effect primarily seen during off-hours with a 28% reduction in median door-to-puncture times.

**Conclusions:** Institutional protocols help diminish the weekend effect in endovascular stroke treatment. This is driven largely by improvement in off-hours performance, with protocol adherence leading to further decreases in treatment intervals over time.

## Introduction

The principal goal of acute, large vessel, ischemic stroke management is rapid reperfusion of the ischemic penumbra. This requires the engagement and coordination of multiple aspects of emergent care. Epidemiologic data reveal worse functional outcomes for stroke patients presenting during off-hours, a phenomenon referred to as the weekend effect (1–4). This has been attributed to non-uniform access to subspecialty services during off-hours. Efforts to ensure optimal outcomes across all times have included implementing institutional intravenous thrombolysis protocols, organizing care around comprehensive stroke centers, and leveraging telemedicine to provide around-the-clock stroke coverage in the absence of local providers (5, 6). In large stroke centers with the capability of providing around-the-clock coverage, these efforts have been effective in negating the weekend effect on intravenous thrombolysis and stroke outcomes (7, 8).

The emergence of endovascular therapy as the standard of care for large-vessel occlusions poses a new challenge to ensuring optimal around-the-clock stroke care. Previous efforts demonstrated the role of quality improvement analysis in identifying and minimizing delays in endovascular stroke therapy and suggested a relationship between shorter door-to-puncture times and presentation during regular work hours (9). This study assesses the impact of institutional protocols for endovascular stroke therapy on the weekend effect.

## Materials and Methods

Data were obtained from a prospectively maintained database of patients receiving mechanical thrombectomy at an academic comprehensive stroke center from June 2012 to October 2015. This study met all HIPAA guidelines and was approved by the Institutional Review Board. Consecutive patients with anterior circulation large vessel occlusions confirmed by angiography and receiving endovascular stroke therapy were included. Baseline clinical and demographic data was prospectively collected. All patients were evaluated by a board-certified neurologist for thrombectomy eligibility.

When appropriate, patients were treated with intravenous thrombolytics per the institutional standard of care. Patients were selected for endovascular stroke therapy per an established institutional stroke protocol (9, 10). A new protocol was instituted in 2012 to help standardize and accelerate treatment times (9, 10). Requirements for treatment included an intracranial proximal artery occlusion (internal carotid artery, middle cerebral artery M1 or proximal M2), NIHSS ≥8, and time last seen well within 8 h of groin access. Procedural sedation was preferred under the protocol, and stentrievers were exclusively used until September 2014 after which aspiration techniques were also employed, either alone or with stentrievers.

Management of acute stroke proceeds through a series of discrete events, critical for patient triage and workflow. Our protocol has previously been published (9) In brief, after recognition of a stroke, our institutional protocol is tailored to facilitate rapid transfer to the radiology department for non-contrast head CT and CT angiography, and typically additional hyperacute MRI. Once a large vessel occlusion is suspected, the neuroendovascular team is activated in parallel to the ongoing emergent patient evaluation (before imaging is performed). We have previously demonstrated that the time between image acquisition and groin puncture was the longest and most variable in-hospital interval. Parallel activation facilitates early mobilization of the interdisciplinary neuroendovascular team, thereby accelerating reperfusion therapy (9). Protocol utilization was implemented by our stroke quality team with widespread dissemination of the protocol and ongoing education, while impact and mobilization data was retrospectively measured in monthly intervals, as a proxy for adherence. Adherence was evaluated through weekly quality assurance meetings and monthly stroke center committee meetings. As part of our clinical protocol, the following clinical events were prospectively registered and subsequently analyzed for latency analysis: ictus, door (presentation to our center), non-invasive imaging, groin puncture, and angiographic reperfusion. Door time was determined either from the emergency room registration time, which reflects the time of ambulance arrival, or, in the case of inpatients (in-house strokes), the time of symptom recognition by hospital staff. The time of reperfusion was time of primary intracranial vessel reperfusion. Procedure duration, the interval from puncture to arteriotomy closure was also recorded.

Patients were grouped and analyzed based on day of the week and time of day of their presentation to the emergency department. Regular hours were defined as 07:00–19:00 h on weekdays. Off-hours were defined as 19:00–07:00 h on weekdays, and 19:00 h on Friday to 07:00 h on Monday on weekends. Angiographic results were graded by the modified Thrombolysis In Cerebral Infarction (mTICI) score. Successful reperfusion was defined as a mTICI score of 2b or 3. Outcome was defined by the modified Rankin Scale (mRS) measured prospectively at 90 days in all surviving patients. A favorable outcome was defined as mRS of 0–2.

Characteristics were compared using chi-square or Fisher test for categorical variables, two-sided *t*-test for continuous variables, and Wilcoxon rank sum for ordinal variables. Wilcoxon rank sum was used for the time intervals, which did not follow a normal distribution. All statistical analysis was performed in R (11).

## Results

A total of 129 consecutive patients from June 2012 to October 2015 were included in the present analysis. All patients underwent endovascular stroke therapy for retrieval of a proximal large vessel occlusion in the anterior circulation. Patients either presented in transfer or directly to our hospital. General anesthesia was utilized in 31/129 (24%) of patients.

**Figure 1:**
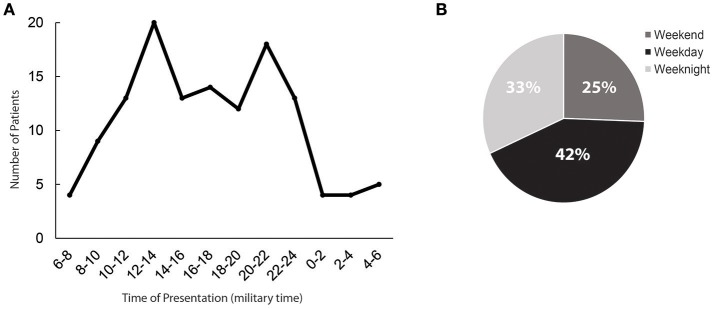
Time of presentation to the emergency department. **(A)** Histogram depiction of the times of presentation, grouped into 2 h windows. Time is shown per a 24-h clock. **(B)** The proportion of patients presenting during on-hours (shown as weekday) vs. those presenting during off-hours (depicted as weeknights and weekends).

54 patients (42%) presented during regular hours, compared to 75 (58%) presenting off-hours; 25% of all presentations occurred on weekends, while 33% presented on weeknights. Presentation frequency was bimodal, peaking at noon and 22:00 h (Figure 1).

All baseline characteristics except for median age and smoking were comparable between the two cohorts, with a trend noted for hyperlipidemia (Table 1); the off-hours cohort was slightly younger (67 vs. 72 years, *p* < 0.02). The proportion of patients with witnessed events, transfer from outside facilities, and utilization of general anesthesia were similar (Table 1). Outcomes between the two groups were not statistically different, including complications, reperfusion rates, discharge disposition and 90-days mortality (Table 1).

**Table 1:** Patient characteristics and outcomes.

**Variable**	**Regular**	**Off-hours**	***p*<**
	***N* = 54**	***N* = 75**	
Age (years)	72 (62–81)	67 (52–78)	0.04
Female	27 (50)	35 (47)	0.9
Atrial fibrillation	23 (43)	31 (41)	1
Diabetes	15 (28)	18 (24)	0.8
Hypertension	43 (80)	53 (71)	0.4
CAD	13 (24)	23 (31)	0.6
Heart Failure	15 (28)	16 (21)	0.6
Hyperlipidemia	28 (52)	25 (33)	0.06
Prior stroke or TIA	12 (22)	13 (17)	0.7
Smoking	16 (30)	8 (11)	0.02
Cancer	10 (19)	7 (9)	0.3
Illicit drug use	3 (6)	4 (5)	1
NIHSS	17 (13–21)	16 (14–19)	0.4
Witnessed stroke	29 (54)	45 (60)	0.6
Outside transfer	10 (19)	14 (19)	0.6
Received IV tPA	32 (59)	50 (67)	0.5
General Anesthesia	15 (18)	16 (21)	0.6
**Outcomes**	**Regular**	**Off-hours**	***p*** **<**
	***N*** **=** **54**	***N*** **=** **75**	
Complications	6 (11)	8 (11)	1
TICI 2b/3	41 (76)	51 (68)	0.5
Discharged home	7 (13)	15 (20)	0.4
mRS at 90 days	3 (2–6)	3 (1–4)	0.3
Good outcome	21 (39)	36 (48)	0.4

*Patient characteristics, angiographic outcome, and functional outcomes compared across hours of presentation. Numbers represent absolute numbers and then percentages (n/total per category) within parentheses except for age, NIHSS, and mRS which are represented by the median with the interquartile range in parenthesis. Good outcome is defined as mRS 0–2 at 90 days. CAD, coronary artery disease; mRS, modified Rankin score; NIHSS, National Institutes of Health Stroke Scale; TIA, transient ischemic attack; tPA, tissue plasminogen activator*.

Median treatment intervals were unrelated to the time of presentation (Table 2). There was increased variability in imaging to puncture and puncture to reperfusion intervals for off-hours presentation, with an interquartile range of 56 vs. 42 min during regular hours and 36 vs. 30 min, respectively. Treatment intervals were decreased compared to historical data from our institution in the period from 2007 to 2011 prior to implementation of a standardized protocol (9) (Table 3).

**Table 2:** Treatment intervals during regular and off-hours.

**Time interval**	**Historical**	**Regular**	**Off-hours**	***p*<**
Ictus to door	–	162 (30–233)	181 (65–237)	0.4
Door to puncture	143 (112–170)	100 (72–123)	94 (71–128)	0.9
Door to imaging	23	21 (14–38)	19 (14–38)	0.5
Imaging to puncture	97 (73–116)	71 (49–91)	71 (44–100)	1.0
Puncture to reperfusion	–	42 (35–65)	48 (29–65)	0.8
Procedure duration		65 (37–83)	64 (51–91)	0.4

*We analyzed each interval between ictus (when known), patient arrival (door), imaging (for primary presentation, groin puncture, vessel opening, and procedure duration. Times are reported as median (in minutes) with interquartile range (IQR). P-values are shown for Wilcoxon rank sum between regular and off-hour groups. Historical data are based on a cohort of 93 patients treated from 2007 to 2011 at our institution prior to protocol implementation*.

**Table 3:** Treatment intervals by quarter.

**Time interval**	**1st Quarter**	**4th Quarter**	***p*<**
Ictus to door	164 (46–232)	186 (52–238)	0.5
Door to puncture	115 (94–136)	94 (68–124)	**0.006**
Regular hours	104 (92–116)	99 (70–125)	0.5
After hours	130 (103–148)	93 (67–117)	**0.002**
Door to imaging	18 (16–38)	18 (14–28)	0.4
Imaging to puncture	83 (67–110)	67 (49–92)	0.09
Puncture to reperfusion	62 (48–106)	36 (25–59)	**0.001**
Procedure duration	71 (54–108)	65 (40–82)	0.3

*Patients were divided into quarters of presentation over the study period. Subgroup analysis is shown for the door to puncture time. Treatment intervals are reported as median in minutes with interquartile range (IQR). P-values are shown for Wilcoxon rank sum test. P-values of less than 0.05 are bolded*.

Most of the patients (84 of 129) underwent hyperacute MRI either immediately following initial CT/CTA, or as an initial imaging study after transfer from an outside hospital. The median door-to-puncture time trended slightly longer in the group that received MRI, 100 (76–124) vs. 86 min (62–127), *p* < 0.09.

The 3-years study period was divided into four consecutive quarters of ~0.75 years, each containing 32 patients, and comparisons were made between the first (Q1) and fourth (Q4) quarters, to assess the impact of increased experience with the protocol (Table 3). There was an improvement in overall median door to puncture times comparing Q1 vs. Q4 (115 vs. 94 min, *p* < 0.006). This improvement was driven predominantly by patients who presented during off-hours, with a 37 min (28%) improvement in median door to puncture times in this group (130 vs. 93 min, *p* < 0.002), with no statistically significant improvement in the group presenting during regular hours (104 vs. 99 min, *p* < 0.5). This improvement was not attributable to significant differences in patient characteristics between Q1 and Q4 (Table 4). There was a progressive shift in our institution from general anesthesia to monitored anesthetic care, reflected in the decreased rates of general anesthesia by Q4. Additionally, puncture to reperfusion times improved from Q1 to Q4 (62 vs. 36 min, *p* < 0.001) although total procedure duration did not change (71 vs. 65 min, *p* < 0.3).

**Table 4:** Patient characteristics by quarter.

**Demographics**	**First *N* = 32**	**Fourth *N* = 32**	***p*<**
Age (years)	71 (63–83)	69 (55–79)	0.4
Female	13 (41)	14 (44)	1.0
Atrial fibrillation	13 (41)	15 (47)	0.9
Diabetes	9 (28)	9 (28)	1.0
Hypertension	22 (69)	27 (84)	0.8
CAD	8 (25)	14 (44)	0.2
Heart Failure	5 (16)	7 (22)	0.7
Hyperlipidemia	17 (53)	13 (41)	0.5
Prior Stroke or TIA	7 (22)	3 (9)	0.3
Smoking	3 (9)	6 (19)	0.5
Cancer	3 (9)	3 (9)	1.0
Illicit Drug Use	2 (6)	0 (0)	0.5
NIHSS	16 (14–19)	17 (13–19)	0.9
Witnessed Stroke	19 (59)	27 (84)	0.06
Outside Transfer	8 (25)	2 (6)	0.1
Received IV tPA	16 (50)	24 (75)	0.08
General Anesthesia	11 (34)	2 (6)	0.02

*Patient characteristics compared across first and last quarters of the series. Except for age and NIHSS, numbers represent absolute numbers and then percentages (n/total per category) within parentheses. For age and NIHSS, numbers represent the median with the interquartile range in parenthesis. CAD, coronary artery disease; NIHSS, National Institutes of Health Stroke Scale; TIA, transient ischemic attack; tPA, tissue plasminogen activator*.

## Discussion

The present study demonstrates that the implementation of acute LVO care protocols mitigated the weekend effect for this highly time-sensitive treatment in this cohort of patients. In previous work, presentation during off-hours was associated with longer door-to-puncture times (9). After protocol implementation, the evaluation times, treatment times, and outcomes, including complication rates and 90 days mRS, were equivalent between patients presenting during regular and off hours and were decreased compared to our published data collected prior to protocol implementation (9). Together, our results extend on recent observations that the weekend effect can be overcome in large stroke centers (12) by demonstrating the role of institutional protocols in standardizing and accelerating care. Further, and uniquely, this study demonstrates that the observed benefits of protocol adherence and familiarity are driven by improvements in off-hours care.

Following recent evidence of powerful benefit from endovascular stroke therapy for LVOs it is essential to evaluate and improve the delivery of such care (13–18). Endovascular management of acute stroke from LVOs requires complex, emergent diagnostic and therapeutic procedures. During off-hours these processes are at risk because of the considerable strain they can place on a healthcare system. Analogs of the weekend effect are recognized in the literature. Specifically, the cardiac experience is instructive, with persistent delays in coronary reperfusion and worsened outcomes for patients presenting with ST-segment elevation myocardial infarction during off-hours (19). Similar delays in reperfusion times have been demonstrated in endovascular stroke therapy, although sample sizes have been underpowered to detect an impact on clinical outcome (20, 21). A subsequent registry review of over 12,000 patients demonstrated that patients presenting to non-teaching hospitals during weekends for endovascular stroke therapy had higher grade eventual disability (22). In teaching hospitals, where coverage was likely more uniform, there was no detectable weekend effect.

This and other analyses have demonstrated that implementing institutional protocols for LVO patient triage and treatment reduced door-to-puncture time (9, 23). Beyond implementation, increasing use of a protocol generates familiarity (9). Increasing familiarity with the protocol was evidenced by door-to-puncture times improving from 2012, when the protocol was relatively new, to the end of 2015, when we analyzed our data. Interestingly, most of the improvement occurred in the patients who presented off-hours, where a 28% reduction in door-to-puncture times was observed. Thus, although there was no statistical weekend effect in the complete 2012–2015 cohort, a prominent driver of this finding was improvement in treatment times in the off-hours group.

We selected this 3-years period for study because our stroke protocol remained unchanged during this time. Our times are comparable to those in recently published literature (17, 18), but most of our patients had two separate acute imaging studies (CT/CTA and MRI) under the protocol (24). We have access to hyperacute MRI located in the emergency department adjacent to the CT scanner, and we used MRI-DWI to select patients with small core infarcts who were thought at the time of protocol creation to be most likely to benefit from endovascular therapy (24). The additional hyperacute MRI was utilized in approximately two thirds of the patients, with a median door-to-puncture time 14 min greater than those receiving just CT/CTA, which partially explains the latencies to treatment observed in our cohort relative to contemporary studies.

In late 2015, following institutional multi-departmental review of the published endovascular stroke data proving beneficial treatment of patients selected by a single CT/CTA approach, we modified our protocol to a single imaging study and a more streamlined process. Current process times are thus much more rapid (averaging 55 mi from door to groin, data not published). This serves to illustrate that protocols must be living documents, maintained and current to remain effective. Further, it bears emphasis that time intervals to evaluate the impact of protocol implementation should be selected to accurately reflect the protocol. For example, onset to reperfusion is a common metric in stroke care but may not be specific for evaluating an institutional protocol because pre-hospital processes, beyond the purview of the institutional protocol, are included.

There was also an improvement in puncture to reperfusion times over the course of the study period which was not clearly explained by the collected parameters. This may relate to changes in technology and technique, including increased familiarity with stentrievers. In 2014, we began using aspiration in addition to stentriever for thrombectomy (25–27). While this technique may facilitate faster time to initial reperfusion, comparison of aspiration and stentriever techniques has shown equivalence in recent literature (27, 28).

Our institutional protocol optimized time saved by parallel activation of the Neuroendovascular team during emergent patient evaluation and treatment decision making. During regular hours the Neuroendovascular team is on-site and team activation is therefore rapid. However, most stroke occurs outside regular working hours (Figure 1), as confirmed in a recent multicenter study evaluating this question (27). To minimize the impact of this off-hours timing, our institutional protocol requires that the Neuroendovascular team is alerted for patients transferring in within the treatment window with a high NIHSS, before imaging is performed. The team is therefore typically already in the hospital preparing the angiography suite when the patient arrives in transfer, leading to significant time savings.

This study has several limitations. First, this is a single-center experience in an academic comprehensive stroke center, potentially limiting the generalizability of our results. A significant portion of patients were transferred from outside facilities for treatment, allowing earlier activation off-hours of the interventional teams who can mobilize while the patient is in transit. The study period starts with the advent of stentrievers but spans multiple milestones in the endovascular management of LVO, including the adoption of aspiration techniques, and publication of randomized control trials demonstrating efficacy for these interventions. While these serve as a potential confounder in patient selection, reperfusion times, or outcomes the principle of protocoled approaches to care is one that remains independent of these factors. Finally, the size of our cohort limits the power to detect small differences in treatment or outcomes between the cohorts. Larger studies are required to detect subtle but real differences in treatment times and outcomes after thrombectomy (22).

The findings of this study demonstrate that the use of institutional protocols can mitigate the weekend effect for patients undergoing endovascular stroke therapy for LVO at centers that receive a substantial proportion of patients in transfer. Additionally, it demonstrates that protocol adherence and familiarity accelerates treatment times, particularly in the off-hours cohort. Together, these findings highlight the importance of establishing institutional and regional protocols to optimize acute stroke management. Replication in larger independent cohorts is necessary to confirm these findings.

## Author Contributions

SR and TL-M designed the study. SR, FA, and TL-M generated primary data, wrote, and critically revised the manuscript. CS, BM, RC, RG, JR, LS, AP, and JH critically revised the manuscript.

### Conflict of Interest Statement

LS reports being the principal investigator of an investigator-initiated study of extended-window intravenous thrombolysis funded by the National Institutes of Neurological Disorders and Stroke (clinicaltrials.gov/show/NCT01282242) for which Genentech provides alteplase free of charge to Massachusetts General Hospital as well as supplemental per-patient payments to participating sites; serving as a scientific consultant regarding trial design and conduct to Lundbeck (international steering committee, DIAS3, 4 trial), Penumbra (data and safety monitoring committee, Separator 3D trial) and Medtronic (Victory AF and Stroke AF trials). AP: Consultant for Medtronic and Penumbra. JH: No relevant conflicts, conflicts outside of this work include consulting for Medtronic and Globus and serving on the DSMB of a trial sponsored by Codman Neuromuscular. He also has a grant from the Harvey L. Nieman Policy Institute and serves as a senior affiliate research fellow. The remaining authors declare that the research was conducted in the absence of any commercial or financial relationships that could be construed as a potential conflict of interest.
